# 

**Published:** 1813-01

**Authors:** 


### MEDICAL AND PHYSICAL JOURNAL.

CONDUCTED BY

SAMUEL FOTHERGILL, M.D.

AND

WILLIAM ROYSTON, ESQ. F.L.S.

VOL. XXIX.

FROM JANUARY TO JUNE, 1813.

Et quoniam variant morbi, variabimus artes;
Mille mali species, mille salutis erunt.

*LONDON:*

PRINTED FOR RICHARD PHILLIPS; BY J. ADLARD, 23, BARTHOLOMEW-CLOSE, AND 39, DUKE-STREET, SMITHFIELD; AND SOLD BY J. SOUTER, NO. 1, PATERNOSTER-ROW.

174225
5135

W
1
ME 182

[Entered at Stationers' Hall.]

### MONTHLY CATALOGUE OF MEDICAL BOOKS.

CALLOW'S Catalogue (Part II. for 1813) of elegant Books, with Plates; Liber Medici, Anatomica, Chirurgici, et Chemici; with an extensive assortment of French, Italian, and German, Works, many of which are recently imported; including a selection of Second-hand English Books, many of them of great rarity.

A Treatise on Worms and other Animals which infest the Human Body, with the most speedy, safe, and pleasant, means of cure. By T. Bradley, M.D. 12mo. plates.—Underwood.

Cursory Remarks on Corpulency. By a Member of the Royal College of Surgeons. The second Edition, with additions, and illustrated by an Engraving.—Callow.

---

### *NOTICES TO CORRESPONDENTS.*

*Mr. Adams's paper, asserting his claim to the discovery of certain curative processes in Diseases of the Eye, will appear in our next Number; it arrived too late for insertion in the present. Mr. Blair's notes on Syphilis, announced last month, have not yet been received, but we have reason to expect the first paper will appear in the ensuing Number of our Journal. Communications have reached us from Dr. Christie, Mr. Davies, Mr. Leath, Medicus, Senex, Juvenis, &c. &c. We are obliged to Mr. Rigby for a copy of his work, to which we shall pay early attention.*

